# A novel 3D in vitro model of the human gut microbiota

**DOI:** 10.1038/s41598-020-78591-w

**Published:** 2020-12-09

**Authors:** Francesco Biagini, Marco Calvigioni, Anna Lapomarda, Alessandra Vecchione, Chiara Magliaro, Carmelo De Maria, Francesca Montemurro, Francesco Celandroni, Diletta Mazzantini, Monica Mattioli-Belmonte, Emilia Ghelardi, Giovanni Vozzi

**Affiliations:** 1 Research Center “E. Piaggio”, University of Pisa, Largo Lucio Lazzarino 1, 55122 Pisa, Italy; 2 Department of Information Engineering, University of Pisa, Via G. Caruso 16, 56122 Pisa, Italy; 3 Department of Translational Research and New Technologies in Medicine and Surgery, University of Pisa, Via San Zeno 37, 56127 Pisa, Italy; 4 Department of Clinical and Molecular Science-DISCLIMO, Università Politecnica delle Marche, Via Tronto 10/A, 60126 Ancona, Italy

**Keywords:** Biotechnology, Tissue engineering

## Abstract

Clinical trials and animal studies on the gut microbiota are often limited by the difficult access to the gut, restricted possibility of in vivo monitoring, and ethical issues. An easily accessible and monitorable in vitro model of the gut microbiota represents a valid tool for a wider comprehension of the mechanisms by which microbes interact with the host and with each other. Herein, we present a novel and reliable system for culturing the human gut microbiota in vitro. An electrospun gelatin structure was biofabricated as scaffold for microbial growth. The efficiency of this structure in supporting microbial proliferation and biofilm formation was initially assessed for five microbes commonly inhabiting the human gut. The human fecal microbiota was then cultured on the scaffolds and microbial biofilms monitored by confocal laser and scanning electron microscopy and quantified over time. Metagenomic analyses and Real-Time qPCRs were performed to evaluate the stability of the cultured microbiota in terms of qualitative and quantitative composition. Our results reveal the three-dimensionality of the scaffold-adhered microbial consortia that maintain the bacterial biodiversity and richness found in the original sample. These findings demonstrate the validity of the developed electrospun gelatin-based system for in vitro culturing the human gut microbiota.

## Introduction

The human gut microbiota consists of more than a thousand taxonomic units and exerts a marked influence on the host during both homeostasis and disease^1^. Contribution to the intestinal protection from pathogens, development of intestinal architecture, and implication in food and drug metabolism are few of the documented functionalities of the human gut microbiota^2^. Particularly, intestinal microorganisms attend to short chain fatty acid (SCFA) production, cofactor and vitamin biosynthesis, bile salts transformation, and production of enzymes able to transform dietary complex polysaccharides into structurally simpler carbohydrates, which otherwise would not be digested or absorbed^3,4^. Furthermore, some bacterial species populating the human gut (e.g. *Lactobacillus brevis* and *Bifidobacterium dentium*^5^) can produce neurotransmitters, such as γ-aminobutyric acid (GABA), noradrenaline, and dopamine^2^. Thus, abnormal changes in the microbiota composition, known as dysbiosis, may have crucial effects on several local and systemic disorders, further supporting the idea that the human gut microbiota can influence human well-being^6–12^.

In the last decades, most of the research efforts on the gut microbiota have been focused on clinical studies or animal models. Unfortunately, there is an intrinsic difficulty to frequently access to the human gut for monitoring microbial composition, metabolite and enzyme production, as well as fermentative or inflammatory processes^13^. As regards other animals, studies on these models can be limited by crucial species-specific differences, involving e.g*.* a different diet and eating frequency^14^. Due to their technical reproducibility, easy access and monitoring^15^, in vitro models represent a valid alternative to study the microbial behavior of the human gut microbiota.

Several attempts have been made in culturing single bacterial strains on synthetic scaffolds. In this context, electrospun structures have already been used to enhance the adhesive capabilities of bacteria^16,17^ given their sub-micrometer structure with a high aspect ratio^18^. *Burkholderia terricola* adhesion on a poly(ε-caprolactone) nanofiber mats was widely studied by De Cesare and colleagues^17^. Also, Anonye and coworkers^19^ co-cultured an intestinal epithelial cell line with *Clostridium difficile* on a polyethylene terephthalate electrospun structure to in vitro evaluate bacterial adhesion and the subsequent tissue infection. Thus, the use of a fibrous scaffold appeared promising being able to mimic the mechanical and physical properties of the native colonized tissue^20^. To date, although some in vitro models were proposed for culturing complex microbial populations as those residing in the gut (e.g. TIM-2^21^, SHIME^22^, Three-Stage Continuous System^23^), no attempts have been made to use electrospun structures as scaffolds for microbial adhesion and growth.

The present work aimed at developing a stable 3D in vitro model of the human gut microbiota by using an electrospun natural polymer-based scaffold. In particular, we characterized the electrospun material for its mechanical and physical properties and analyzed its applicability as scaffold for culturing the human fecal microbiota. The ability of the electrospun gelatin structures to preserve the typical bacterial biodiversity and richness of the fecal microbiota was also investigated.

## Results

### Mechanical and physical characterization of the scaffolds

The electrospinning process is a widely used technique to create microporous structures^24^. Here, this technique was used to biofabricate an electrospun structure made of a solution of gelatin crosslinked with (3-Glycidoxypropyl)-trimethoxysilane (GPTMS). The resulting scaffold was characterized for its mechanical and physical properties.

**Figure 1:**
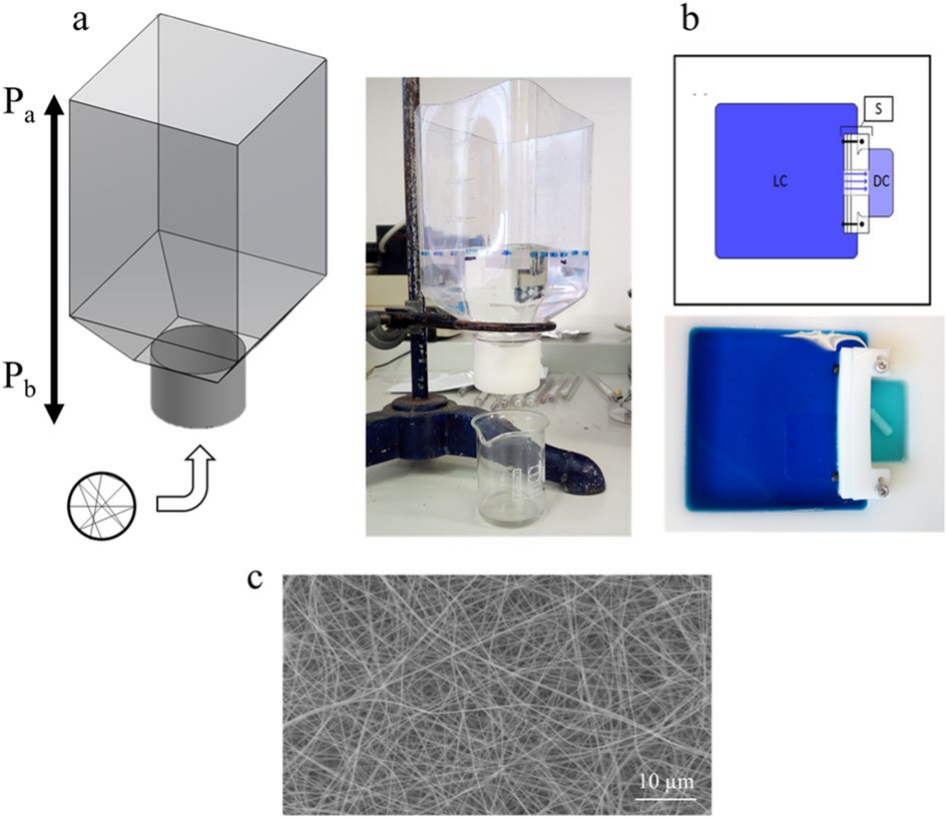
Mechanical and physical characterization of the electrospun gelatin structure **(a)** Permeability test set-up. P_a_ represents the water pressure and P_b_ the atmospheric pressure. **(b)** Diffusion test set-up. The large chamber (LC) was filled with methylene blue and the diffusion chamber (DC) with deionized water. Reproduced with permission^25^. Copyright 2015, *Journal of Biomedical Materials Research*. **(c)** SEM image of the electrospun gelatin structure (× 2000 magnitude).

Mechanical properties were evaluated by using a tensile test. The average elastic modulus in dry and in wet conditions was 23.75 $\pm$ 2.58 MPa and 199.46 $\pm$ 44.26 kPa, respectively. The permeability coefficient was calculated through the Darcy formula by using the experimental set-up of Fig. 1a and was equal to (5.37 ± 0.07) × 10^–14^ m^2^. Diffusion across the membrane was evaluated with the model developed by Montemurro et al.^25^ (Fig. 1b) by using methylene blue dye and was equal to (2.53 ± 0.13) × 10^–8^ m s^−2^. The average diameter of the fibers constituting the scaffold was estimated through scanning electron microscopy (SEM) imaging (Fig. 1c). The results indicated a diameter of 0.58 $\pm$ 0.18 μm. The fibers were homogeneously distributed within the scaffold and randomly oriented with no preferential alignment or evident beads. The thickness of the electrospun gelatin structures was 0.198 $\pm$ 0.032 μm.

### Growth of single strains on the electrospun scaffolds

**Figure 2:**
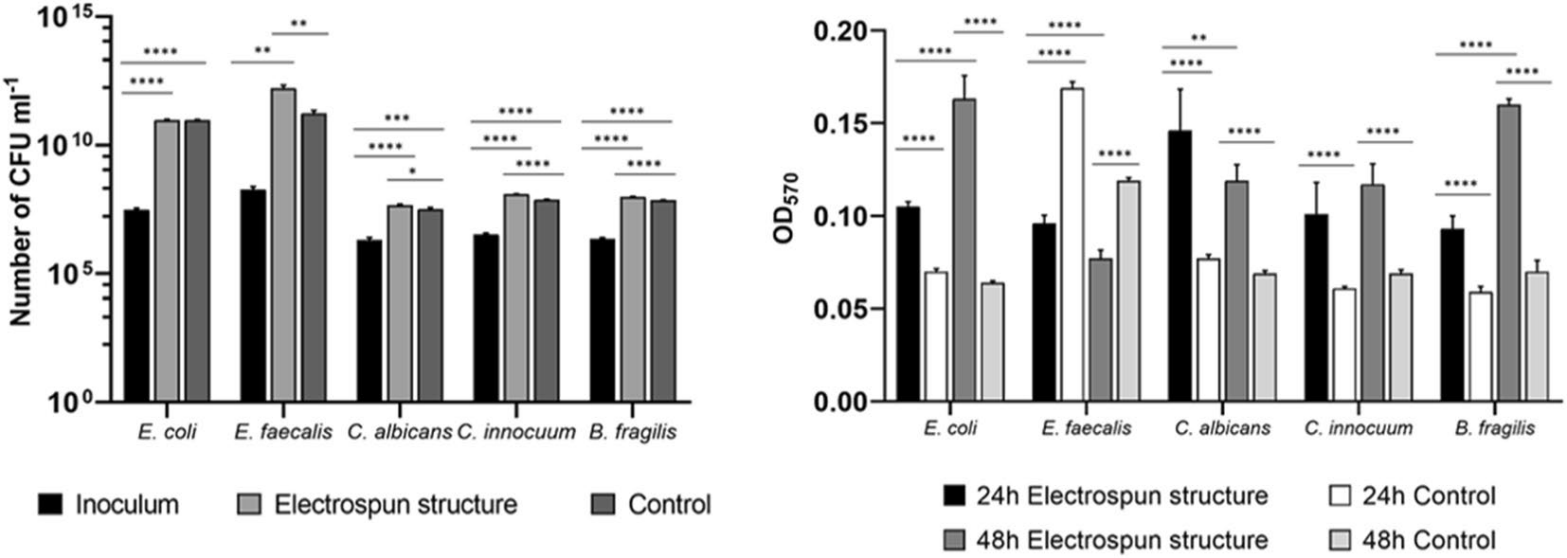
Growth and biofilm formation by *E. coli*, *E. faecalis*, *C. innocuum*, *B. fragilis*, and *C. albicans* on the electrospun gelatin scaffolds. **Left:** Microbial counts (number of CFUs ml^-1^) at the inoculum (black bars) and after 24 h of incubation on the electrospun gelatin structures (grey bars) and on controls (i.e. glass slides, dark grey bars); **Right:** microbial biomass after 24 and 48 h of incubation with and without (control) the electrospun structures. * p < 0.05, ** p < 0.01, *** p < 0.001, **** p < 0.0001.

The electrospun gelatin structures were tested for their ability to preserve viability and support microbial growth in RPMI 1640 medium. *Escherichia coli*, *Enterococcus faecalis, Clostridium innocuum, Bacteroides fragilis,* and *Candida albicans* were selected for the analyses since common inhabitants of the human gut. Glass slides without the gelatin structures were used as controls. After a 24-h incubation, the number of colony-forming units (CFUs) on each support was determined. As shown in Fig. 2a, the number of microorganisms grown on the scaffolds was significantly higher at 24 h compared to the initial inoculum (p < 0.01). The number of collected *E. coli* cells was almost identical on the electrospun structures and on the control slides. On the other hand, the CFU number of *E. faecalis*, *C. albicans, C. innocuum,* and *B. fragilis* was higher on the gelatin structures than on controls (*E. faecalis* p < 0.01, *C. albicans* p < 0.05, *C. innocuum* p < 0.0001, *B. fragilis* p < 0.0001). These results demonstrate that the electrospun structures are suitable for microbial viability and growth.

Since adhesion to biotic or abiotic surfaces is essential for planktonic microorganisms to develop stable sessile populations, crystal violet assay for biofilm quantification was performed to evaluate microbial ability to form long-lasting multilayered consortia. The ability of five microbial strains to adhere and form biofilm communities on the electrospun gelatin structures and on glass slides was evaluated after 24 h and 48 h of incubation (Fig. 2b). *E. coli*, *C. albicans, C. innocuum,* and *B. fragilis* showed a significant increase in biomass when cultured on the electrospun structures when compared to controls after 24 h and 48 h of incubation (p < 0.0001, Fig. 2b). *E. faecalis* acted conversely since the biofilm biomass was lower on the scaffolds than on controls after 24 h and 48 h of incubation (p < 0.0001). This behavior could be linked to the intrinsic properties of gelatin, which is a hydrophilic and natural polymer strongly different from the hydrophobic glass slide used as positive control. Significant differences between the amount of biofilm formed on the scaffolds at 24 h and 48 h were observed. The amount of biofilm produced by *E. faecalis*, *C. albicans,* and *B. fragilis* was higher at 24 h (*E. faecalis* p < 0.0001, *C. albicans* p < 0.01, *B. fragilis* p < 0.0001). On the contrary, *E. coli* biomass was more abundant at 48 h (p < 0.0001). No statistically significant difference between *C. innocuum* biomasses at 24 h and 48 h was found.

Taken together, these results highlight the efficiency of the electrospun gelatin structures in maintaining microbial viability, sustaining growth, and promoting biofilm formation by model gut microorganisms.

### In vitro-cultured microbiota on gelatin scaffolds

**Figure 3:**
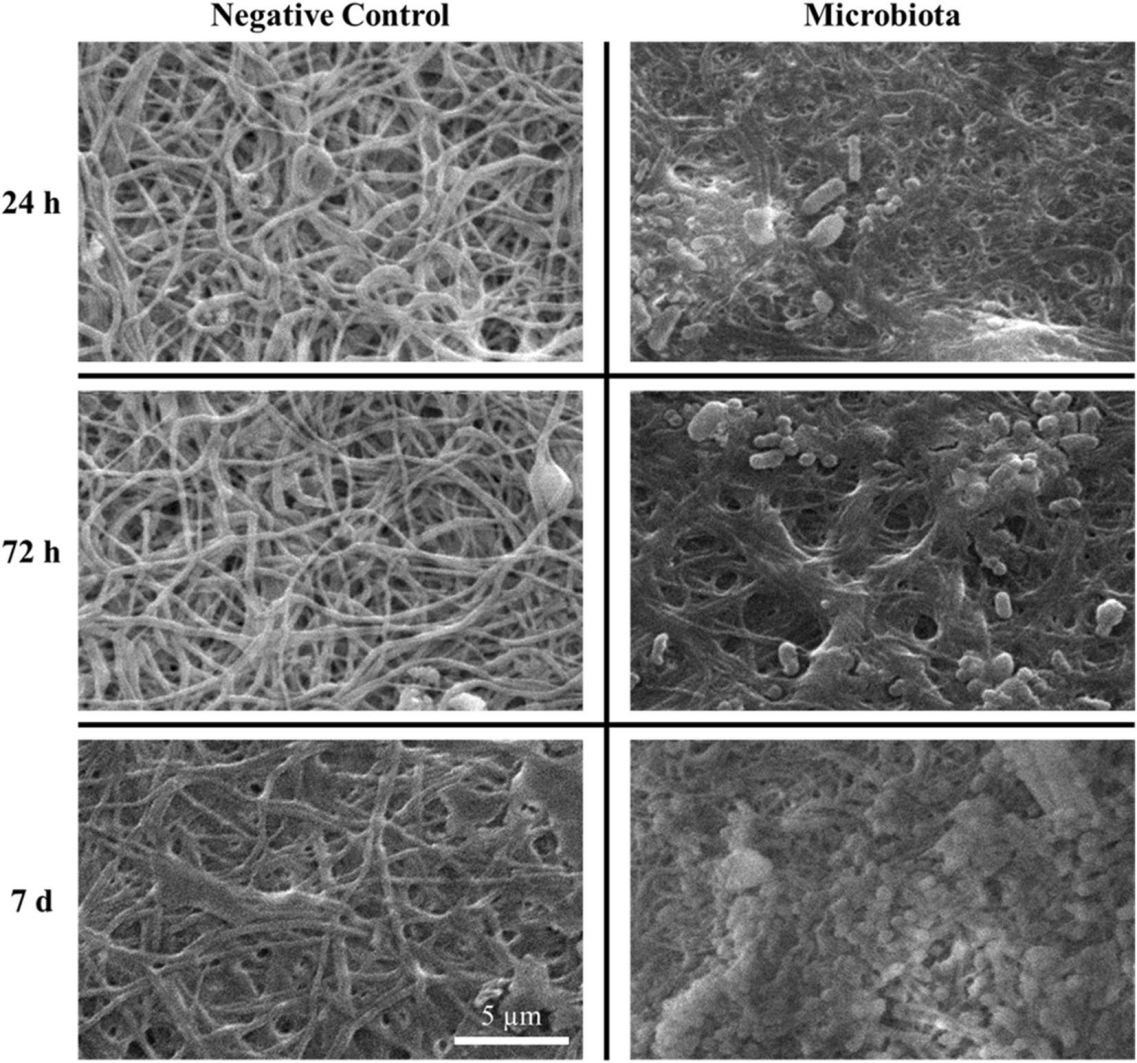
Electron microscopy of electrospun scaffolds without microbes (negative controls) and with the fecal microbiota (× 4000 magnification) at 24 h, 72 h, and 7 days post-inoculation.

**Figure 4:**
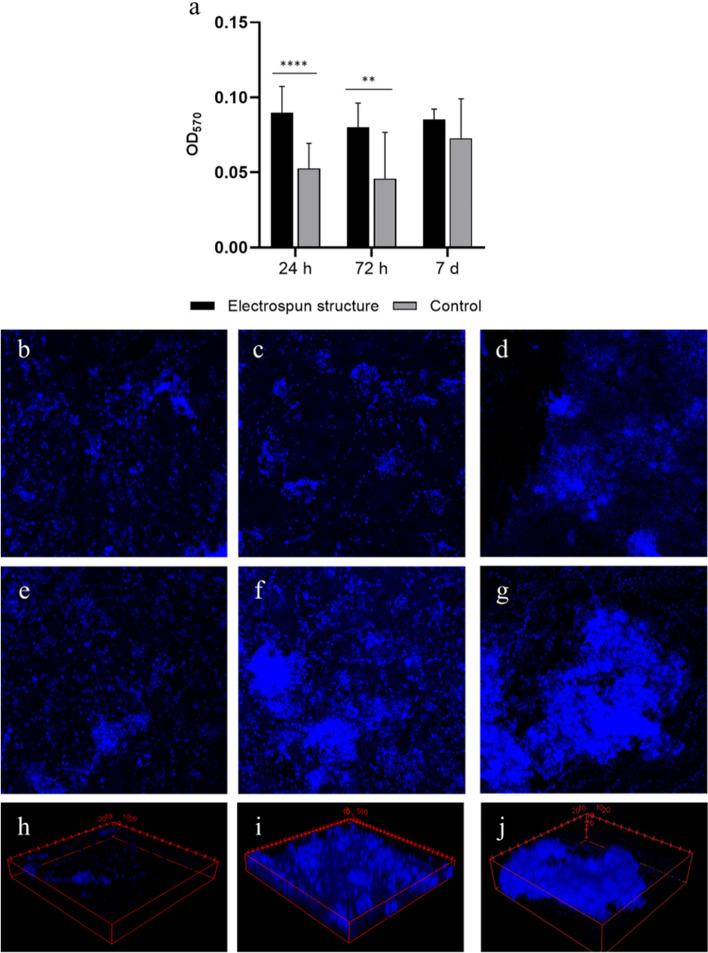
Analysis of the in vitro-cultured microbial biomasses. **(a)** Crystal violet quantification of microbial consortia at 24 h, 72 h, and 7 days of incubation on the scaffolds (black bars) and on glass slides (control, grey bars). ** p < 0.01, **** p < 0.0001; **(b–d)** visualization of the DAPI-stained biomasses at 24 h **(b)**, 72 h **(c)**, and 7 days **(d)** of incubation on glass slides (× 10 magnification); **(e–g)** Z-stack visualization of the DAPI-stained biomasses at 24 h **(e)**, 72 h **(f)**, and 7 days **(g)** of incubation on the electrospun scaffolds (×10 magnification); **(h–j)** 3D visualization of the DAPI-stained biomasses on the electrospun structures at 24 h **(h)**, 72 h **(i)**, and 7 days **(j)** post inoculation. The three-dimensional development of a biofilm that expands over the entire thickness of the structure is visible.

The fecal microbiota was prepared according to the European Guidelines for fecal microbiota transplantation^26^ and incubated in RPMI 1640 medium on the electrospun structures and on glass slides (controls) for 24 h, 72 h and 7 days at 37 °C. Figure 3 shows the growth of the fecal microbiota on scaffolds at different time points. The in vitro-cultured microbiota biomass on the gelatin scaffolds and on the control slides was measured by the crystal violet assays at each time point (Fig. 4a). The biofilm biomass was significantly more abundant on the electrospun scaffolds than on controls after 24 h (p < 0.0001) and 72 h (p < 0.01) of incubation, while no statistically significant difference was highlighted at day 7 post-inoculation (Fig. 4a). Confocal electron microscopy of DAPI-stained bacteria showed that the fecal microbiota adhered to the gelatin structures (Fig. 4e–g; control slides Fig. 4b–d) and formed a three-dimensional, multi-layered stable biofilm that persisted for at least 7 days (Fig. 4h–j). These data indicate that the electrospun gelatin structures are more suitable than glass slides for supporting adhesion and growth of the fecal microbiota, especially in the early stages of in vitro culture.

### rRNA 16S sequencing and metagenomic analysis

**Figure 5:**
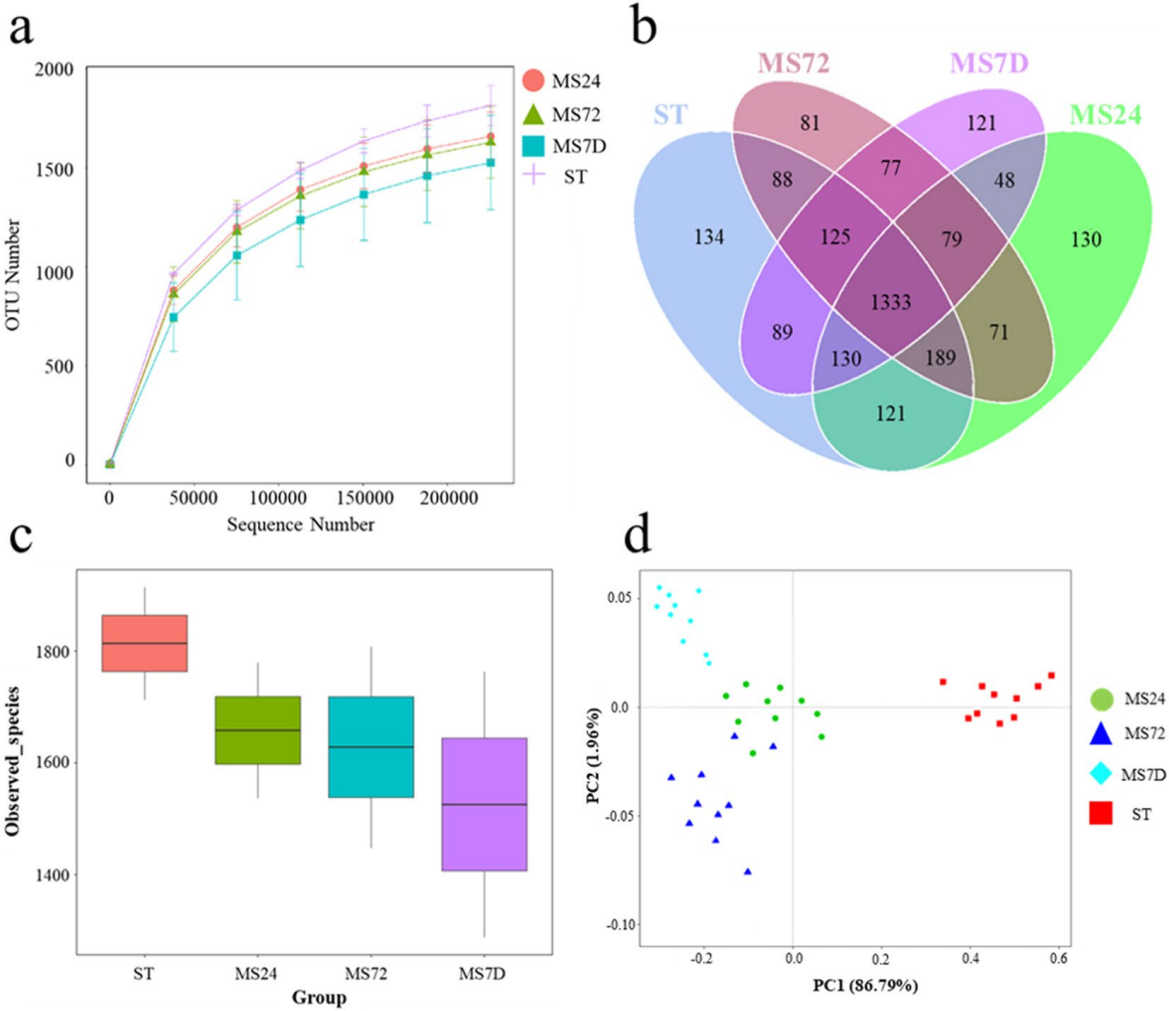
Metagenomic analysis of the fecal microbiota and of the microbial populations after incubation on the scaffolds. **(a)** Rarefaction curves for OTU number in the fecal microbiota (ST) and in those cultured on the electrospun gelatin structures (24 h: MS24; 72 h: MS72; 7 d: MS7D); **(b)** Venn diagram shows the number of shared OTUs between the fecal microbiota (ST) and the cultured ones at different times (24 h: MS24; 72 h: MS72; 7 d: MS7D) by the overlap; **(c)** comparison of the observed microbial species between the fecal microbiota (ST) and after incubation (24 h: MS24; 72 h: MS72; 7 d: MS7D) on the electrospun gelatin structures; **(d)** Principal coordinates analysis (PCoA) visualization of the weighted Unifrac distances between the microbial communities.

To evaluate the performance of the gelatin structures in maintaining the gut microbial biodiversity, the electrospun scaffolds were inoculated with fecal microbiota samples and the composition of the microbial communities grown in vitro was analyzed. Purified DNA samples were subjected to metagenomic analysis to compare the distribution and relative abundance of microbial consortia in the original fecal samples and on the electrospun gelatin structures at different incubation times (24 h, 72 h, and 7 days)^27^. Bacterial biodiversity and richness inside the four different groups (ST: fecal microbiota; MS24, MS72, and MS7D: microbiota samples cultured in vitro for 24 h, 72 h, and 7 days, respectively) were estimated by rarefaction curves performed by randomly sampling more than 20.000 times with replacement and calculating the total number of Operational Taxonomic Units (OTUs) identifiable from these sequenced samples. The rate of acquisition of new OTUs in fecal microbiota samples paralleled with the acquisition of new OTUs in cultured samples, suggesting equal levels of bacterial richness in all groups (Fig. 5a). 1333 OTUs were maintained following the in vitro cultivation of the microbiota on the electrospun structures at different time points (Fig. 5b) and no significant differences were observed in the number of detected species among the four groups (Fig. 5c). Beta diversity analysis indicated that after 24 h, 72 h, and 7 days of growth on the scaffolds, the distance between in vitro-grown samples was consistently lower than the distances between each of these and the original fecal sample (Fig. 5d).

**Figure 6:**
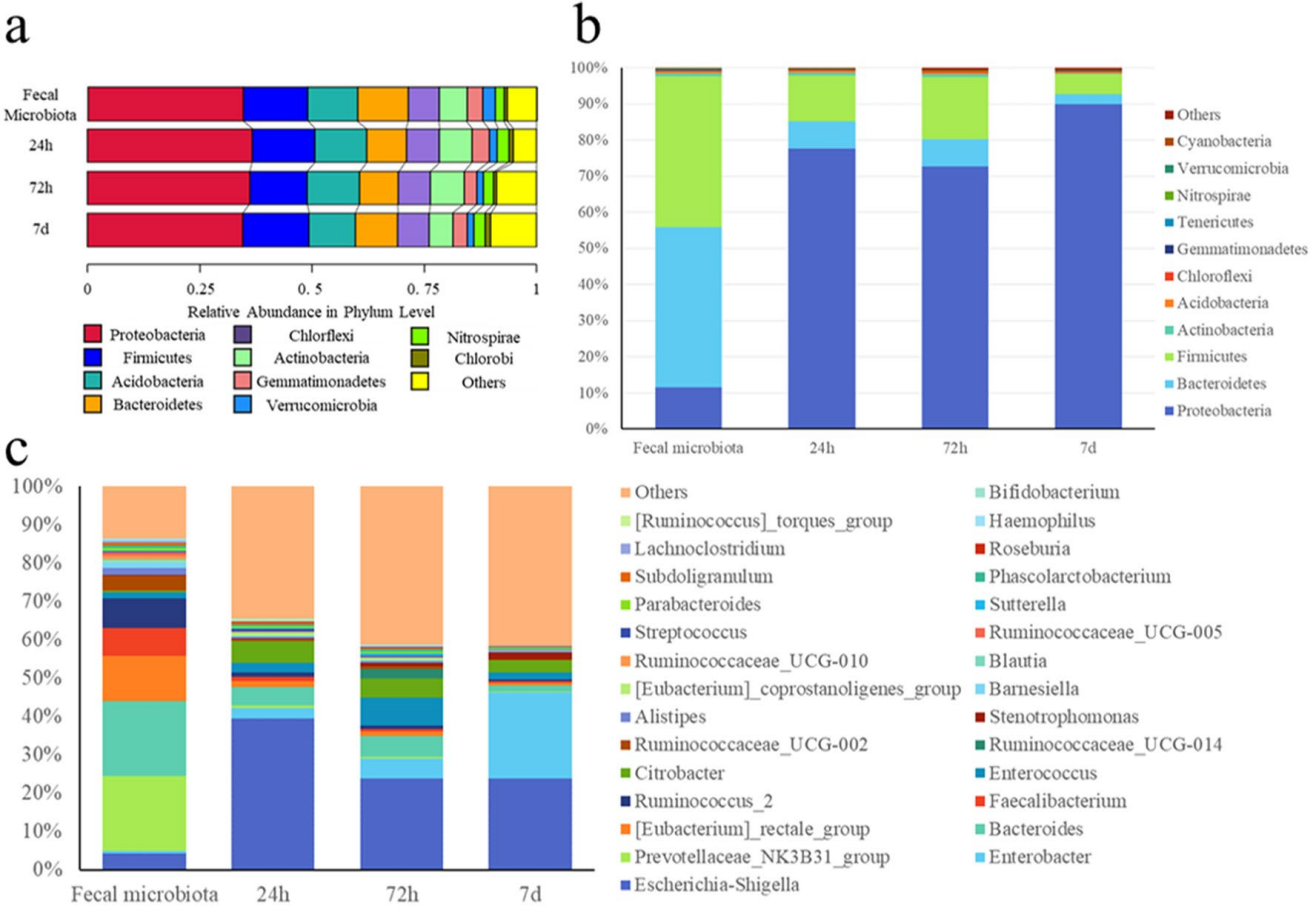
Metagenomic analysis of the fecal microbiota and of the microbial populations after incubation on the scaffolds: bacterial relative abundances. **(a)** *Phyla* plotted on unweighted Unifrac distances metrics; **(b)** species relative abundance in *phylum*; **(c)** species relative abundance in *genus*.

All the *phyla* forming the original fecal microbiota were preserved on the scaffolds for up to 7 days, even the less abundant and relevant ones, such as *Verrucomicrobia*, *Nitrospirae,* and *Chlorobi* (Fig. 6a). Relative abundances were calculated to evaluate the distribution of the main *taxa* in each group of samples. Culture on the electrospun structures resulted in certain fluctuations at *phylum* and *genus* level. As shown in Fig. 6b, the relative abundance of *Bacteroidetes*, *Firmicutes*, and *Proteobacteria* differed between fecal samples and in vitro-cultured samples, with an expansion in the number of *Proteobacteria* starting from 24 h of incubation on the scaffolds. As far as *genera* concern, major fluctuations were observed with *Escherichia-Shigella*, *Enterobacter*, and *Citrobacter*, *whose* relative abundances increased if compared to the fecal microbiota. *Bacteroides*, *Prevotellaceae_NK3B31_group*, and *Faecalibacterium* behaved conversely (Fig. 6c).

Taken together, data demonstrate that in vitro culture of the fecal microbiota on the electrospun structures ensures the maintenance of all the *phyla* present in the fecal sample, as well as biodiversity and richness of the bacterial consortia, despite reasonable fluctuations of certain *taxa*.

### Real-time quantitative PCR (real-time qPCR)

**Figure 7:**
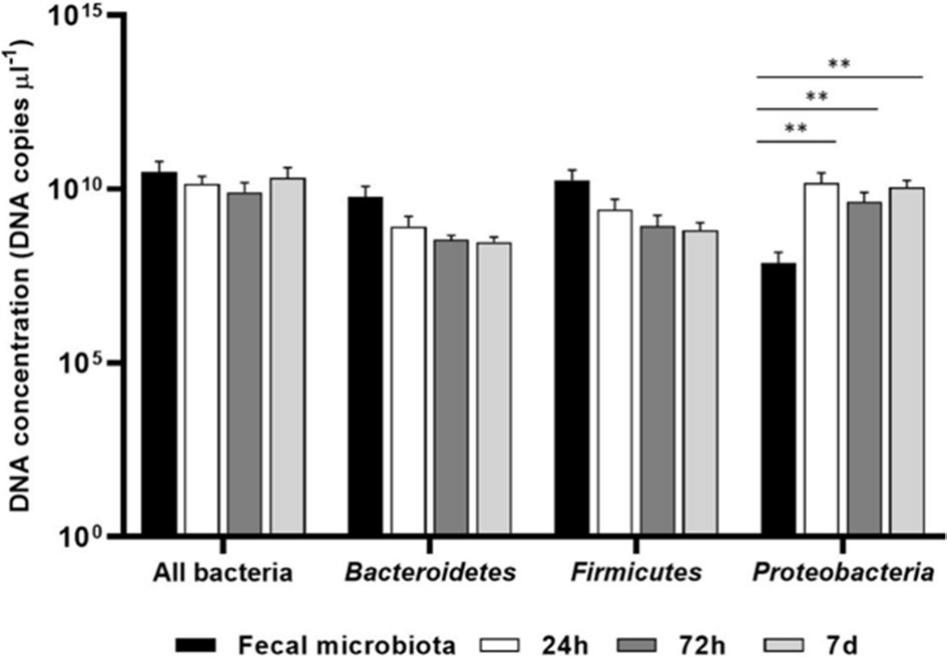
Absolute quantification of microbial populations performed by Real-Time qPCRs. DNA concentration (DNA copies µl^-1^) relative to the 16S rRNA gene for total bacterial load (all bacteria), *Bacteroidetes*, *Firmicutes*, and *Proteobacteria* in fecal microbiota (black bars) and in vitro-cultured microbiota at 24 h (white bars), 72 h (grey bars), and 7 d (light grey bars). ** p < 0.01.

Extracted DNA was also subjected to Real-Time qPCR using specific primer pairs (see supporting information (SI), Table S2) to quantify the amount of total bacteria and of bacteria in selected *phyla* (i.e. *Bacteroidetes*, *Proteobacteria*, *Firmicutes*). As shown in Fig. 7, the number of DNA copies µl^−1^ for all bacteria was comparable in the fecal microbiota and in the in vitro cultures on the scaffolds at each time point (24 h, 72 h, 7 days). Similarly, no differences in the amount of *Bacteroidetes*, as well as of *Firmicutes*, were found. A significant increase in the number of DNA copies µl^−1^ for *Proteobacteria* was observed starting from 24 h of incubation on the gelatin structures in comparison with the fecal microbiota (p < 0.01).

Taken together, our findings indicate that incubation on the scaffolds does not alter the total bacterial load, as well as the amount of *Bacteroidetes* and *Firmicutes*, while stimulates *Proteobacteria* expansion. The selection of this *taxon* is in agreement with the metagenomic results, which report *E. coli* as the main cause of this numerical increase.

## Discussion

Despite the efforts of the scientific community, witnessed by initiatives such as the Human Microbiome Project^28^, the role of microbial flora in influencing hosts’ health and disease is still unclear^29–31^. Even if in vivo animal models are traditionally used to monitor possible variations in the gut microbiota and its interplay with the host, they are strongly limited by their low translational power into clinical outcomes^32,33^. For this reason, the intuition that the human gut microbiota can be studied by using in vitro models has arisen. In fact, since 1981 when Miller and Wolin designed their semi-continuous fermenter^34^, dynamic in vitro models were used to roughly simulate the complexity of the intestinal environment and to improve the knowledge on the composition of the inhabiting microbial communities in response to different stimuli^22,23,35–37^.

In this study, we present a biofabricated electrospun gelatin structure that, due to its mechanical and physical properties, can be applied to many biological contexts, including the development of in vitro culture models of the human gut microbiota.

Electrospinning was chosen since already used as biofabrication technique to reproduce the gut micro-environment^38,39^. Several papers demonstrate that the random and submicrometric structure, produced during the electrospinning process, promotes bacterial colonization^17,40,41^. By SEM and confocal laser microscopy, we demonstrated that the human fecal microbiota forms a stable biofilm with a three-dimensional microbial disposition on the surface and among the fibers of the scaffolds at different times of culture (Figs. 3, 4e–j). These data are corroborated by the observation that the electrospun gelatin structures are significantly better than glass slides in ensuring the viability of three control microbial strains. In addition, biofilm formation by *E. coli*, *C. albicans*, *C. innocuum*, *B. fragilis,* and the fecal microbiota is sustained and enhanced by the presence of the electrospun gelatin structures for at least 7 days of culture. Most likely, both the reticular and gelatinous nature of this support guarantees microbial success in constituting such a multi-layered architecture, which is not present on glass control slides.

Although some works in the literature refer to only hydrophobic surfaces to sustain the adhesion of different bacterial strains^16,42^, our results suggest that also a hydrophilic substrate as gelatin may promote cell adhesion and biofilm formation. Moreover, adhesive proteins produced by bacteria, such as adhesins, could help the attachment of the microorganisms on biological and natural surfaces such as gelatin. These findings show that not only the surface wettability can influence microbial attachment, but also the chemical properties of the surface^43^.

Losing biological richness is probably the main problem associated with culturing microbiota inhabiting all body districts, not only the intestinal one^44,45^. Many bacterial species co-evolved together with their human host and they often fail in surviving and multiplying far from their specific ecological niche. The construction of a three-dimensional reticular structure that can reproduce the complex pattern of bacterial interactions characterizing the human gut, may facilitate microbial survival after removal from the human host. Considering the standardized in vitro-culture conditions used in this study, the finding that the microbial biodiversity is preserved almost in its integrity for 7 days of culture exceeded our expectations. Certainly, qualitative and quantitative fluctuations in the microbial composition are unavoidable during in vitro culturing and are observable in all the taxonomic ranks from the *phylum* to the species level. The quantitative expansion in the *phylum Proteobacteria* detected by qPCR is mainly attributable to an increase of *E. coli*, whose growth is enhanced in the used culture conditions than other species. Even slightly changing the culture conditions, we could probably observe a completely different situation. It would be of great interest to investigate how variations in temperature, culture medium, partial pressure of oxygen, or other factors modulate the microbiota composition when grown on the electrospun gelatin structures.

In conclusion, we demonstrate the efficiency of these innovative structures in supporting microbial adhesion and growth, as well as their applicability as three-dimensional substrates for culturing the human gut microbiota in vitro. To the best of our knowledge, this is the first report demonstrating the real possibility to culture the human gut microbiota on electrospun gelatin scaffolds, and assessing the production of stable and long-lasting biofilms, as well as the maintenance of the microbial community composition with only little fluctuations during the culture period.

The technical feasibility, versatility, and high reproducibility confer a great potential to this in vitro model. For example, it can be used to perform in-depth analyses concerning gut microbiota composition and production of metabolites and how these parameters are modulated in response to different factors (e.g. nutrients, drugs, probiotics, infecting agents). In addition, the inclusion of this model in co-culture dynamic devices with human cells (e.g. bioreactors or microfluidic devices) would also open the way to new studies aimed at deciphering the countless interactions that occur between intestinal microorganisms and the host, in healthy or pathological conditions in a personalized perspective.

## Materials and methods

### Fabrication of the electrospun gelatin structures

Gelatin structures were produced by electrospinning a 10% w/v solution of gelatin (type A from porcine skin, Sigma-Aldrich, Italy) in 9:1 (v/v) glacial acetic acid (99.8%, Sigma-Aldrich) and demineralized water. After complete dissolution, 3.68% v/v GPTMS ((3-Glycidoxypropyl)-trimethoxysilane) (97%, Alfa Aesar, Germany) was added to the solution as gelatin crosslinker and mixed at room temperature (RT) for 40 min. The gelatin-based solution was electrospun using an electrospinning apparatus (Linari, Italy) for 1 h under an applied DC voltage of 30 kV and a feeding rate of 1.2 ml/h. A distance of 10 cm between metal needle (21 gauge) and collector was used. Electrospun structures were left to dry over a week at RT for reaching complete solvent evaporation.

### Mechanical and physical characterization

Tensile tests were conducted using a Zwick/Roell mod Z005 equipped with a 100 N load cell. All the tests were performed in both dry and wet (i.e. totally immersed in deionized water) conditions. The tensile experiments were performed on samples with width x gauge lengths of 1 cm × 8 cm. Initial grip-to-grip separation of about 4 cm and a cross-head speed of 0.1 mm^−1^ were used. The mean thickness of each structure was evaluated using a micrometer with a precision of 10 μm, averaging three measures taken at different points of the structure. For each specimen, a stress–strain curve was obtained and the elastic modulus derived.

Permeability coefficient was evaluated using the experimental setup shown in Fig. 1a. Briefly, we fixed the electrospun structure on the bottom of the bottle (inserted on a special cap, with a bore in its central part) and we filled the bottle with deionized water. Using this setup, the permeability was calculated through the Darcy formula (Eq. 1):

1 $$Q=\frac{kA}{\mu }\frac{{P}_{a}-{P}_{b}}{L}$$

where Q is the flow rate (expressed in m^3^ s^−1^), P_a_ and P_b_ the pressures of the fluid and the atmosphere across the gelatin structure, respectively (expressed in Pa), L the thickness of the gelatin structure (expressed in m), μ the dynamic viscosity of the fluid (expressed in Pas), A the area of the structure in contact with the fluid (expressed in m^2^) and k the permeability coefficient (expressed in m^2^). The flow rate was evaluated by measuring the volume of water that passes through the membrane in 20 min. The level of water inside the bottle was maintained the same during the experiment, to keep constant the pressure drop across the gelatin structure.

The diffusion coefficient across the electrospun gelatin structure was evaluated using the experimental setup described by Montemurro et al.^25^ and shown in Fig. 1b. Briefly, the electrospun gelatin structure was placed in the hole separating the large chamber (LC) and the diffusion chamber (DC). Methylene blue (Sigma, Italy) was used as a reference molecule for the test. The LC chamber was loaded with 150 ml of a 25 mg l^−1^ solution of methylene blue while the other chamber with 15 ml of deionized water. To evaluate the diffusion coefficient, we used the equation described by Montemurro (Eq. 2):

2 $$D=\frac{L}{A}\frac{{V}_{1}{V}_{2}}{{V}_{1}+{V}_{2}}\frac{1}{t}\mathrm{ln}\left(1-\frac{\left({V}_{1}+{V}_{2}\right){C}_{2}}{{V}_{1}{C}_{1}}\right)$$

where L is the thickness of the gelatin structure (expressed in m), A the area of the hole between the two chambers (expressed in m^2^), V_1_ and V_2_ the volumes of the two chambers, respectively (expressed in m^3^), C_1_ and C_2_ the concentrations (expressed in mol m^-3^) and t the time (expressed in s). Different aliquots were taken from the diffusion chamber at different time points and the concentration was measured using FLUOstar Omega spectrofluorimeter (BMG Labtech, Germany) at wavelength of 244 nm.

### Microbial strains and microbiota preparation

For the biological preliminary tests, five microbial strains were selected as typical commensals of the human gut microbiota: *Escherichia coli* (ATCC 25922), *Enterococcus faecalis* (ATCC 29212), *Bacteroides fragilis* (clinical isolate), *Clostridium innocuum* (clinical isolate), and *Candida albicans* (ATCC 10231). These microorganisms were maintained as stocks at − 80 °C until use.

Potential stool donors (< 60 years) underwent a medical interview to exclude history of gastrointestinal, neurological, and metabolic disorders, as well as associated risk factors. For each donor, a blood sample and a stool sample were screened for infectious diseases 4 weeks before donation, according to the recent European Guidelines for fecal microbiota transplantation^26^. No recent exposure (< 3 months) to antibiotics, immunosuppressants, and chemotherapy was also mandatory. Considering these exclusion criteria, a single healthy donor was selected. Fecal samples were collected and processed anaerobically within 6 h after defecation^26^. Briefly, 30 g of fresh feces were dissolved in 150 ml of 0.9% w/v NaCl, filtered with sterile gauzes to remove the larger corpuscular particles, analyzed to ensure the absence of pathogenic microorganisms by multiplex PCR amplifications (FilmArray GI Panel, Biomérieux, France), and stored at − 80 °C in 10% v/v glycerol.

### Microbial growth on the scaffolds

Electrospun gelatin structures were cut and overlaid onto 30 mm diameter glass cover slips (Thermo Fisher Scientific, Germany). Glasses with the structures were inserted into wells of 6-well microplates (Corning, USA). The gelatin electrospun structures were sterilized by using 2 ml of 70% v/v ethanol (Sigma-Aldrich) solution and incubating for 15 min in a sterile environment. Ethanol was removed and wells exposed to UV light for 15 min in a sterile environment. 2 ml of RPMI 1640 culture medium (Sigma-Aldrich) were added to each well. Control wells were prepared by using glass cover slips as substrate for microbial culture. Sterility control wells (negative controls) containing the electrospun scaffolds and the medium, but without the addition of microorganisms, were also included.

*E. coli*, *E. faecalis*, *C. innocuum*, *B. fragilis*, and *C. albicans* stocks were thawed, streaked on blood agar plates (Biomérieux), and grown at 37 °C for 24–48 h. Isolated colonies were inoculated in 5 ml of RPMI 1640 medium (Sigma-Aldrich). Suspensions were incubated at 37 °C for 24 h and aliquots of 100 µl were inoculated in each well of the microplate prepared as described above. Microplates were incubated at 37 °C and adhesion assays were conducted at 24 h and 48 h post-inoculation. At the inoculum and after 24 h of incubation, 100 µl aliquots of the microbial suspensions were serially diluted and seeded on blood agar plates. Plates were incubated at 37 °C for 24–48 h and the number of CFUs ml^-1^ determined. Fecal microbiota samples were thawed and 100 µl aliquots were used to inoculate multi-well plates as described above. Plates were incubated at 37 °C in anaerobic atmosphere by using Oxoid AnaeroGen (Thermo Fisher Scientific) for a total of 7 days. Every 72 h, 670 µl of the medium were replaced with an equal volume of fresh medium. For each time point (24 h, 72 h, and 7 days post-inoculation), adhesion assays, confocal laser and scanning electron microscopy observations, Real-Time qPCRs, and metagenomic analyses were carried out.

### Biofilm biomass measurement

To evaluate the adhesion of microorganisms to the electrospun gelatin structures, the crystal violet assay was performed^46^. The culture medium was removed and non-adherent planktonic microorganisms were eliminated by washing wells three times with 1 ml of PBS. Microbial biofilms were stained by using 2 ml of 0.1% w/v crystal violet (Carlo Erba, Italy) for 30 min at RT^46^. The wells were washed three times with 1 ml of deionized water and covered with 2 ml of absolute ethanol for 15 min at RT to solubilize the crystal violet. Aliquots of ethanol-crystal violet solution (200 μl) were transferred to a 96-wells plate and the optical density at 570 nm (OD_570_) was measured by using a microplate reader (Biorad model 550, Biorad, USA)^47,48^. The absorbance was adjusted by subtracting the mean OD_570_ of the sterility controls to the obtained OD_570_ of each sample.

### Confocal laser and scanning electron microscopy

After supernatant removal, wells were washed three times with 1 ml of PBS. Microorganisms were fixed by adding 1 ml of 2% w/v paraformaldehyde (PFA, Sigma-Aldrich) and incubating at 4 °C for 16 h in a dark room. After PFA removal, wells were washed three times with 1 ml of PBS. For confocal laser microscopy, fixed samples were stained by adding DAPI (1 μg ml^−1^ in PBS) to each well in a dark room. After a 4-h incubation, DAPI was removed and wells covered with 1 ml of PBS. Plates were stored at 4 °C until microscopic examination. Images were acquired by using a Nikon A1 confocal microscope equipped with a 10 × objective.

For SEM, fixed samples, taken at the different time points (24 h, 72 h, and 7 days) were covered with a solution of 2.5% v/v glutaraldehyde (Sigma-Aldrich) in PBS, post-fixed in 1% w/v osmium tetroxide (Sigma-Aldrich), and dehydrated in increasing ethanol concentrations and hexamethyldisilazane (HMDS) (Sigma-Aldrich). For the acquisition, samples were mounted on aluminum stubs, gold-sputtered by the Edwards Sputter Coater B150S equipment and observed with a Philips XL 20 SEM microscope (FEI Italia SRL, Milan, Italy) at 4000 × magnification. Images from negative controls were also acquired.

Images from both confocal and SEM imaging were analyzed by using ImageJ software (NIH, USA) and a representative result is shown.

### DNA extraction

QIAamp PowerFecal Pro DNA Kit and QIAamp DNA Mini Blood and Tissue Kit (Qiagen, Germany) were used to extract genomic DNA from fecal samples and from wells (supernatants + membranes) at 24 h, 72 h, and 7 days post-inoculation. The extraction procedure was performed following the manufacturer’s protocol. The DNA concentration was calculated by measuring the optical density at 260 nm (OD_260_) and DNA purity was estimated by determining the OD_260_/OD_280_ and OD_260_/OD_230_ ratio with the spectrophotometer BioPhotometer D30 (Eppendorf, Germany).

### Metagenomic analysis

Sequencing and data analysis were carried out by Novogene (Beijing, China). Different regions (i.e. V4, V3–V4, V4–V5) of the gene encoding the 16S rRNA were amplified with specific primers (Supplementary Table S1) by using the Phusion High-Fidelity PCR Master Mix (New England BioLabs, USA). PCR products were purified with the QIAGEN Gel Extraction Kit (QIAGEN) and libraries were generated with the NEBNext UltraTM DNA Library Prep Kit for Illumina and quantified via Qubit and qPCR. The amplicon sequencing was carried out on the HiSeq Illumina platform. Obtained sequences were grouped into OTUs by 97% DNA sequence similarity, which is considered to gather homologous species. Sequence analysis was carried out by using the Uparse software. For each sequence, the Mothur software was run against the SSU-rRNA database of the SILVA Database (http://www.arbsilva.de/) to get the annotations about all the taxonomic ranks (*i.e.* kingdom, *phylum*, class, order, family, genus, species). Phylogenetic relations between all OTUs were determined by using the program of multiple comparison MUSCLE. From the clustering results, alpha and beta diversity were calculated.

### Real-time quantitative PCR (qPCR)

16S rRNA gene-based qPCR analysis of extracted DNAs was used to quantify the amount of total bacteria and of the main *phyla* constituting the fecal microbiota (i.e. *Firmicutes*, *Proteobacteria*, and *Bacteroidetes*) in the original fecal sample and in the samples obtained by incubating the stool for 24 h, 72 h, and 7 days on the electrospun gelatin structures. Primers used in this study are listed in Supplementary Table S2. Primer pairs were chosen to separately detect the amount of *Firmicutes*, *Proteobacteria*, and *Bacteroidetes* in each sample based on the *phylum*-specific region of the 16S rRNA gene^49,50^. A universal primer pair based on the conserved region of the 16S rRNA gene was used to quantify the total amount of bacteria in the samples^51^. qPCR reactions were performed by using the CFX96 Real-Time System (BioRad). All reactions were carried out in duplicate in a 96-wells plate with a final reaction volume of 20 µl, containing 2 µl of 2.5 ng µl^−1^ DNA template, 10 µl of Luna Universal qPCR Master Mix (New England BioLabs), 0.5 µl of each primer (0.25 µM) and 7 µl of sterile water. Two wells with 20 µl of sterile water were used as negative controls. The amplification conditions were as follows: an initial denaturation step at 95 °C for 1 min, followed by 45 cycles of denaturation at 95 °C for 15 s, annealing at primers optimal temperature for 30 s (Table S2), and extension at 72 °C for 10 s. To check the amplification specificity, a melting curve analysis was carried out by increasing the annealing temperature from 65 °C to 95 °C after qPCRs. Serial tenfold dilutions of external standards with known concentration ranging from 10^1^ to 10^12^ DNA copies µl^−1^ were used to generate calibration curves for bacterial quantification in the samples. For each standard curve, the coefficient of determination (R^2^) was greater than 0.98. Absolute quantification was performed by using the CFX Manager Software (Biorad).

### Statistical analysis

Experiments were repeated three times in separate days and data are expressed as the mean ± standard deviation. All the statistical analyses were performed with GraphPad Prism 8 (GraphPad Software Inc., USA). Statistical significance was set at a p-value of < 0.05. For experiments relative to single microbial strains, one-way analysis of variance (ANOVA) followed by Tukey’s multiple comparisons test was performed. For qPCRs, ANOVA followed by Dunnett’s post-hoc correction was applied. The statistically significant difference in the fecal microbiota adhesion assays was ensured by applying the Student t-test for unpaired data.

## Supplementary Information

Supplementary Tables.
