# Supplementary material for: A novel 3D in vitro model of the human gut microbiota
Source: Sci Rep. 2020 Dec 9;10:21499. doi: 10.1038/s41598-020-78591-w (PMC7725811; doi:10.1038/s41598-020-78591-w)
Supplement: Supplementary file 1 — Supplementary Tables. [file 41598_2020_78591_MOESM1_ESM.docx]

**A Novel 3D *In Vitro* Model of the Human Gut Microbiota**

Francesco Biagini^1,2,+^, Marco Calvigioni^3,+^, Anna Lapomarda^1,2^, Alessandra Vecchione^3^, Chiara Magliaro^1,2^, Carmelo De Maria^1,2^, Francesca Montemurro^1,2^, Francesco Celandroni^3^, Diletta Mazzantini^3^, Monica Mattioli-Belmonte^4^, Emilia Ghelardi^3*^ and Giovanni Vozzi^1,2*^

^1^ Research Center “E. Piaggio” - University of Pisa, Largo Lucio Lazzarino 1, 55122, Pisa, Italy

^2^ Department of Information Engineering - University of Pisa, Via G. Caruso 16, 56122, Pisa, Italy

^3^ Department of Translational Research and New Technologies in Medicine and Surgery - University of Pisa, Via San Zeno 37, 56127 Pisa, Italy

^4^ Department of Clinical and Molecular Science - DISCLIMO Università Politecnica delle Marche, Via Tronto 10/A, 60126, Ancona, Italy

^+^ These authors contributed equally to the work.

*emilia.ghelardi@med.unipi.it

*g.vozzi@ing.unipi.it

**Supporting information**

Table S1. Primers used for metagenomic analyses.

| Region | Amplicon length (bp) | Primer name and sequence (5’-3’) |
| --- | --- | --- |
| Bacterial 16S V4 | 292 | \| 515F, \| GTGCCAGCMGCCGCGGTAA \| \| --- \| --- \| \| 806R, \| GGACTACHVGGGTWTCTAAT \| |
| Bacterial 16S V3-V4 | 466 | \| 341F, \| CCTAYGGGRBGCASCAG \| \| --- \| --- \| \| 806R, \| GGACTACNNGGGTATCTAAT \| |
| Bacterial 16S V4-V5 | 393 | \| 515F, \| GTGCCAGCMGCCGCGGTAA \| \| --- \| --- \| \| 907R, \| CCGTCAATTCCTTTGAGTTT \| |

Table S2. Primers used for Real-Time qPCRs.

| Investigated bacterial group | Primer name and sequence (5’-3’) | Amplicon length (bp) | Annealing temperature (°C) | Reference |
| --- | --- | --- | --- | --- |
| Total bacterial load | Eub338F, ACTCCTACGGGAGGCAGCAG  Eub518R, ATTACCGCGGCTGCTGG | 200 | 60 | ^51^ |
| *Bacteroidetes* | Bact934F, CATGTGGTTTAATTCGATGAT  Bact1060R, AGCTGACGACAACCATGCAG | 126 | 60 | ^50^ |
| *Firmicutes* | Firm934F, ATGTGGTTTAATTCGAAGCA  Firm1060R, AGCTGACGACAACCATGCAC | 126 | 60 | ^50^ |
| *Proteobacteria* | Eco1457F, CATGACGTTACCCGCAGAAGAAG  Eco1652R, CTCTACGAGACTCAAGCTTGC | 195 | 63 | ^49^ |
